# Relationship between Kidney Dysfunction and Ischemic Stroke Outcomes: Albuminuria, but Not Estimated Glomerular Filtration Rate, Is Associated with the Risk of Further Vascular Events and Mortality after Stroke

**DOI:** 10.1371/journal.pone.0155939

**Published:** 2016-05-23

**Authors:** Seung-Jae Lee, Dong-Geun Lee

**Affiliations:** Department of Neurology, Sejong General Hospital, Bucheon, Gyeonggi-do, South Korea; Emory University, UNITED STATES

## Abstract

**Background and Objective:**

Estimated glomerular filtration rate (eGFR) and albuminuria are known to be associated with ischemic stroke outcomes. In this study, we investigated the longitudinal relationships of the two markers with mortality, vascular events and functional outcomes in a stroke cohort.

**Methods:**

A total of 295 patients with acute ischemic stroke were prospectively recruited in a single center between May 2012 and February 2015. Renal dysfunction was defined as a decreased eGFR (<60 mL/min/1.73 m^2^) or albuminuria (urine albumin-to-creatinine ratio ≥ 30 mg/g). Good functional outcome at 6 months was defined as a modified Rankin scale score ≤ 2, and the occurrence of major vascular events (stroke, acute coronary syndrome or peripheral artery occlusion) or death was monitored. The associations between renal dysfunction and mortality, major vascular events, and 6-month functional outcome were evaluated by the Cox proportional hazards model and logistic regression analysis. Unadjusted and adjusted hazards ratios (HRs), odds ratios (ORs), and 95% confidence intervals (CIs) were obtained. A Kaplan–Meier survival curve for composite adverse events (major vascular events or death) was also computed according to the presence or absence of albuminuria.

**Results:**

Albuminuria, not eGFR, was significantly associated with mortality (*P* = 0.028; HR 2.15; 95% CI 1.09–4.25) and major vascular events (*P* = 0.044; HR 2.24; 95% CI 1.02–4.94) in the multivariate Cox proportional hazards models adjusting for age, sex, diabetes, hypertension, current smoking, atrial fibrillation, previous stroke, alcohol history, initial National Institutes of Health Stroke Scale (NIHSS) score and eGFR. In addition, albuminuria was negatively associated with 6-month functional outcome in the multivariate logistic regression analysis adjusting for age, sex, diabetes, hypertension, current smoking, atrial fibrillation, previous stroke, alcohol history and eGFR (*P* = 0.001; OR 0.36; 95% CI 0.20–0.65), but the association disappeared when NIHSS score was additionally adjusted (*P* = 0.519; OR 0.79; 95% CI 0.39–1.60). Furthermore, the patients with albuminuria had a significantly higher rate of composite adverse events than the patients without albuminuria (*P* < 0.001 by log-rank test).

**Conclusions:**

Albuminuria seems a more useful clinical indicator than eGFR in evaluating the risk of adverse outcomes including further vascular events and death in patients with ischemic stroke.

## Introduction

Chronic kidney disease is a valuable predictor of adverse outcomes including mortality in patients who suffer ischemic stroke [1–7] as well as in the general population [8–9]. General renal status can be roughly judged by serum creatinine level but more accurately evaluated from estimated glomerular filtration rate (eGFR), which is usually automatically calculated in a clinical setting, based on serum creatinine and basic demographic findings (age, sex, and ethnic group) [10, 11]. Proteinuria (or albuminuria) is also a marker of renal impairment. The urinary albumin-to-creatinine ratio (UACR) calculated using spot urine is recommended for screening the presence of kidney damage in adult patients with cardiovascular disease, as routine dipstick is not sensitive enough to detect “microalbuminuria” [11, 12]. In fact, albumin can be normally detected at small amounts (< 30 mg/day), but albuminuria above a particular level signifies a failure of glomerular filtration or tubular reabsorption of albumin [12, 13]. Thus, eGRF and albuminuria have been used as diagnostic biomarkers for renal disease in clinical practice.

Several studies have investigated the relationships of the two biomarkers with mortality or poor functional recovery after stroke [3–7]. Most of these studies indicate that albuminuria is more closely associated with the adverse outcomes after stroke than eGFR [3–6]. In addition, other studies examined their association with incident stroke in a chronic kidney disease population [13] and a national general population [14]. The results of the studies also showed that albuminuria, but not eGFR, was significantly associated with incident stroke. However, there is no study to our knowledge which compares the contributions of the two renal biomarkers to both mortality and further vascular events after stroke.

Accordingly, we investigated the longitudinal relationships of the two biomarkers with mortality, vascular events and functional outcome in our stroke cohort.

## Methods

### Ethics Statement

This is a prospective observational study which had no possibility of doing harm the health of participants. The study was approved by the Institutional Review Board of Sejong General Hospital. The written consent for the study was obtained from patients or their legal guardians.

### Patients and Clinical Assessment

A consecutive cohort of 408 patients with acute ischemic stroke or transient ischemic attack (TIA) within 7 days from symptom onset was prospectively recruited when admitted to the neurovascular or cardiovascular center of our hospital for the stroke between May 2012 and February 2015. We excluded 82 patients with no urine albumin data (including 12 patients undergoing dialysis), six with significant neurologic sequelae from a previous stroke (score 3–5 on modified Rankin scale [mRS]; 0 = No symptoms at all; 1 = No significant disability despite symptoms: able to carry out all usual duties and activities; 2 = Slight disability: unable to carry out all previous activities, but able to look after own affairs without assistance; 3 = Moderate disability: requiring some help, but able to walk without assistance; 4 = Moderately severe disability: unable to walk without assistance and unable to attend to own bodily needs without assistance; 5 = Severe disability: bedridden, incontinent and requiring constant nursing care and attention; 6 = Dead) [15], and 25 patients with no 6-month outcome data. Finally, 295 patients were included.

All included patients underwent 1.5-T magnetic resonance imaging (MRI) on admission. MRI consisted of diffusion-weighted, gradient echo, and fluid-attenuated inversion recovery images, as well as three-dimensional time-of-flight intracranial MR angiography and contrast-enhanced MR angiography including the extracranial carotid and vertebral arteries.

Clinical information included age, sex, history of hypertension (defined as use of antihypertensive agent before admission, systolic pressure > 140 mmHg, or diastolic pressure > 90 mmHg demonstrated on repeated examinations at least 1 month after presentation with a stroke), diabetes mellitus (defined as fasting blood glucose > 126 mg/dl or history of treatment for diabetes mellitus), hyperlipidemia (defined as total cholesterol level > 200 mg/dl or low density lipoprotein cholesterol > 130 mg/dl at the time of presentation or a history of treatment), current cigarette smoking, heavy alcohol consumption (>26 Soju drinks/month; about 20% alcohol), history of stroke, valvular heart disease, atrial fibrillation, or ischemic heart disease (defined as a known history or clinical demonstration of myocardial infarction or angina pectoris), medication use (anthrombotics, statin, and angiotensin receptor blocker/angiotensin-converting enzyme inhibitor) for ≥ 3 months at stroke onset, and the National Institutes of Health Stroke Scale (NIHSS) score at admission [15].

### Evaluation of Renal Dysfunction and Follow-Up

Serum creatinine was measured within 24 hours after admission. eGFR was calculated using serum creatinine and the Chronic Kidney Disease Epidemiology Collaboration equation adjusted for Asians [10]. Urine samples were collected within 72 hours after admission. Urine albumin was detected by immunoturbidimetric assay using a Roche/Hitachi Modular P analyzer (Hitachi, Tokyo, Japan). UACR was estimated in mg albumin/g creatinine (mg/g). Renal dysfunction was defined as a decrease in eGFR (<60 mL/min/1.73 m^2^) or albuminuria (UACR ≥ 30 mg/g) [11].

All survivors were followed up at the outpatient clinic or by telephone interview. We tried to contact relatives of patients who were lost to follow-up to collect information on the patients’ condition including daily living activities. We monitored further major vascular events (stroke, TIA, acute coronary syndrome, or peripheral artery occlusion) or death. Composite adverse events were defined as major vascular events or death. Good functional outcome at 6 months was defined as mRS score ≤ 2. The nature of the vascular event was preferentially based on medical records of the attending physician. If these were not available, the information was acquired by a telephone interview with the patient, their relatives, or attending healthcare providers in other institutions.

### Statistical Analysis

Statistical analyses were performed with SPSS ver. 18.0 software (SPSS Inc., Chicago, IL, USA). The independent *t*-test or chi-square test was used to compare differences between the patient groups with and without renal dysfunction. Then, eGFR was categorized into three groups: eGFR ≥ 60 (reference), 45 to < 60, and < 45 mL/min/1.73 m^2^. In addition, albuminuria was initially classified into three groups according to previous reports: UACR < 30 (reference), 30 to < 299 (microalbuminuria), and ≥ 300 mg/g (macroalbuminuria) [6, 12]. However, only 13 patients had macroalbumnuria, so the study patients were dichotomized into patient groups of UACR < 30 and ≥ 30 mg/g. The longitudinal associations between renal dysfunction and mortality or major vascular events were evaluated by univariate and multivariate Cox proportional hazards models. The relationship between renal dysfunction and 6-month functional outcome (mRS ≤ 2) was assessed by logistic regression analysis. Age, sex, diabetes mellitus, hypertension, current smoking, atrial fibrillation, previous stroke, alcohol history, and NIHSS score were adjusted in the multivariate analysis. In the multivariate model for the albuminuria, the eGFR was additionally adjusted. In the multivariate model for the eGFR, the albuminuria was additionally adjusted.

Unadjusted and adjusted hazards ratios (HRs), odds ratios (ORs), and 95% confidence intervals (CIs) were obtained. A Kaplan–Meier survival curve for composite adverse events was computed according to the presence or absence of albuminuria (UACR ≥ 30 or < 30 mg/g). Differences in outcomes were estimated using the log-rank test. *P*-values < 0.05 were considered significant.

## Results

The mean age of the 295 patients (157 males and 138 females) was 67.6 years (range, 14–94 years) at admission. Of the 295 patients, 130 (44.1%) had significant albuminuria (UACR ≥ 30 mg/g), and 56 (19.0%) had a decreased eGFR (35 with eGFR of 45 to < 60 mL/min/1.73 m^2^ and 21 with eGFR < 45 mL/min/1.73 m^2^). The significant albuminuria was detected in 40.2, 60.0 and 61.9% of the patients with eGFR of ≥60, 45 to < 60 and <45 mL/min/1.73 m^2^, respectively (linear by linear association test, *P* = 0.008).

Table 1 shows the clinical characteristics of the patients with and without renal dysfunction. Patients with a decreased eGFR (<60 mL/min per 1.73 m^2^) were older and had a higher frequency of female, hypertension, antithrombotics and statin use, while the patients with normal eGFR had a higher prevalence of smoking and alcohol histories. In addition, patients with a significant albuminuria (UACR ≥ 30 mg/g) were older, and had a higher score of NIHSS and a higher prevalence of atrial fibrillation and valvular heart disease. Use of antithrombotics was so significantly associated with history of atrial fibrillation (66.3% versus 41.6%, *P*< 0.001) and previous stroke (70.7% versus 46.5%, *P* = 0.004); statin use was closely associated with age (70.5±10.5 versus 66.5±14.8 yr, *P* = 0.012), diabetes (37.3% versus 22.6%, *P* = 0.010) and hypertension (30.8% versus 14.9%, *P* = 0.007).

**Table 1:** General characteristics of the 295 study patients with and without renal dysfunction; mean ± SD, number (%).

	eGFR (mL/min per 1.73 m^2^)			UACR (mg/g)		
	<60 (N = 56)	≥60 (N = 239)	*P*	≥30 (N = 130)	<30 (N = 165)	*P*
Age (yr)	76.8±9.1	65.4±14.0	<0.001	70.1±12.8	65.6±14.5	0.006
Female gender	38 (67.9)	100 (41.8)	<0.001	64 (49.2)	74 (44.8)	0.454
Hypertension	48 (85.7)	173 (72.4)	0.038	94 (72.3)	127 (77.0)	0.359
Diabetes	21 (37.5)	62 (25.9)	0.083	43 (33.1)	40 (24.2)	0.094
Hyperlipidemia	32 (57.1)	119 (49.8)	0.322	66 (50.8)	85 (51.5)	0.899
Current smoking	3 (5.4)	72 (30.1)	<0.001	27 (20.8)	48 (29.1)	0.103
Previous stroke	9 (16.1)	32 (13.4)	0.601	18 (13.8)	23 (13.9)	0.982
Ischemic heart disease	15 (26.8)	43 (18.0)	0.136	20 (15.4)	38 (23.0)	0.101
Atrial fibrillation	24 (42.9)	74 (31.0)	0.089	54 (41.5)	44 (26.7)	0.007
Valvular heart disease	15 (26.8)	41 (17.2)	0.098	32 (24.6)	24 (14.5)	0.029
Alcohol history	1 (1.8)	45 (18.8)	0.002	18 (13.8)	28 (17.0)	0.463
eGFR	44.6±13.6	93.8±18.7	<0.001	80.7±29.2	87.4±23.4	0.033
UACR	244.1±616.9	73.4±172.4	<0.001	221.0±450.4	15.0±20.6	<0.001
Previous medication						
antithrombotics	42 (75.0)	105 (43.9)	<0.001	67 (51.5)	80 (48.5)	0.603
Statin	25 (44.6)	54 (22.6)	0.001	29 (22.3)	50 (30.3)	0.124
ARB or ACEI	26 (46.4)	86 (36.0)	0.147	51 (39.2)	61 (37.0)	0.691
NIHSS at admission	6.5±7.4	5.0±7.1	0.161	8.1±9.3	3.1±3.7	<0.001

SD, standard deviation; eGFR, estimated glomerular filtration rate; UACR, urinary albumin-to-creatinine ratio; ARB, angiotensin receptor blocker; ACEI, angiotensin converting enzyme inhibitor; NIHSS, National Institutes of Health Stroke Scale.

Median follow-up period was 22.0 months (range, 0.1–39 months). Eight patients died during hospitalization (two heart failure, one septic shock, and five stroke progression) and 41 patients died after discharge (11 unknown causes, seven heart failure, two myocardial infarction, six aspiration pneumonia, eight stroke recurrence, five cancer, one septic shock, and one suicide). Major vascular events occurred in 30 patients (27 strokes, including 26 cases of ischemic stroke and one of subarachnoid hemorrhage, three acute coronary syndrome; two ST-segment elevation myocardial infarctions, and one non-ST-segment elevation myocardial infarction). Fifty-one patients were not being followed up by our clinic at the time of this study. The latest physical condition of 39 of these patients was ascertained by telephone interview. Twelve patients were lost to follow-up because of inability to make telephone contact and were censored at their last clinic visit.

**Fig 1:**
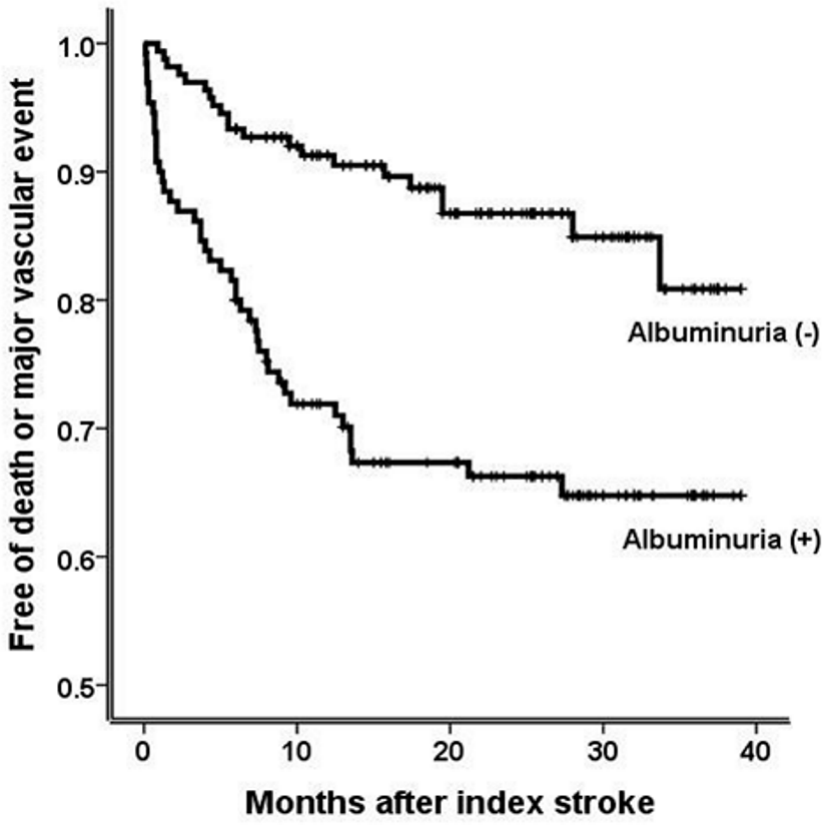
Kaplan–Meier curves for composite adverse events (mortality or major vascular events). The patients with albuminuria (urine albumin-to-creatinine ratio ≥ 30 mg/g) had a significantly higher rate of composite adverse events than the patients without albuminuria (*P* < 0.001 by log-rank test).

Table 2 shows the Cox proportional hazards models for mortality, stroke and major vascular events. In the analysis for eGFR, the level of < 45 mL/min/1.73 m^2^ was associated with mortality in the univariate analysis, but the association disappeared, just showing a statistical trend toward an increase in mortality in the multivariate analysis. In addition, stroke and major vascular events were not associated with the eGFR level. In contrast, albuminuria was significantly associated with mortality and major vascular events in both univariate and multivariate analyses, though it was proved to be associated with stroke only in the univariate analysis. Moreover, a significant difference in composite adverse events (death or major vascular events) was detected on the Kaplan–Meier curve between patients with and without albuminuria (Fig 1).

**Table 2:** Cox proportional hazards models for mortality, stroke, and major vascular events.

			Univariate		Multivariate*	
	Variables		HR (95% CI)	*P*	HR (95% CI)	*P*
Mortality	eGFR	≥60	1.00 (reference)		1.00 (reference)	
		45 to <60	1.37 (0.61–3.09)	0.451	0.72 (0.30–1.72)	0.461
		<45	3.27 (1.51–7.07)	0.003	2.27 (0.96–5.37)	0.062
	UACR	<30	1.00 (reference)		1.00 (reference)	
		≥30	3.42 (1.84–6.36)	<0.001	2.15 (1.09–4.25)	0.028
Stroke	eGFR	≥60	1.00 (reference)		1.00 (reference)	
		45 to <60	2.15 (0.86–5.35)	0.101	1.36 (0.51–3.66)	0.538
		<45	0.74 (0.10–5.54)	0.772	0.61 (0.08–4.85)	0.639
	UACR	<30	1.00 (reference)		1.00 (reference)	
		≥30	2.43 (1.11–5.31)	0.026	2.13 (0.92–4.92)	0.076
Major vascular event	eGFR	≥60	1.00 (reference)		1.00 (reference)	
		45 to <60	1.86 (0.76–4.56)	0.177	1.17 (0.45–3.08)	0.747
		<45	0.65 (0.09–4.78)	0.669	0.52 (0.07–4.06)	0.531
	UACR	<30	1.00 (reference)		1.00 (reference)	
		≥30	2.48 (1.18–5.22)	0.016	2.24 (1.02–4.94)	0.044

HR, hazard ratio; CI, confidence interval; eGFR, estimated glomerular filtration rate (mL/min/1.73 m^2^); UACR, urinary albumin-to-creatinine ratio (mg/g)

*Age, sex, diabetes, hypertension, current smoking, atrial fibrillation, previous stroke, alcohol history, and initial National Institutes of Health Stroke Scale score were adjusted. In the multivariate model for the UACR (albuminuria), the eGFR was additionally adjusted. In the multivariate model for the eGFR, the UACR was additionally adjusted.

Table 3 shows the results of logistic regression analysis for 6-month functional outcome. eGFR was not significantly associated with a good 6-month outcome (mRS ≤ 2). Albuminuria was negatively associated with a good functional outcome in the first adjusted analysis, but the association disappeared when NIHSS score was additionally adjusted.

**Table 3:** Logistic regression analysis for good 6-month outcomes (modified Rankin scale score 0–2).

		Univariate		Multivariate*	
Variables		OR (95% CI)	*P*	OR (95% CI)	*P*
eGFR	>60	1 (reference)		1.00 (reference)	
	45 to <60	0.58 (0.28–1.22)	0.152	1.38 (0.60–3.22)	0.451
	<45	0.69 (0.26–1.78)	0.437	1.86 (0.66–5.26)	0.243
UACR	<30	1 (reference)		1.00 (reference)	
	≥30	0.34 (0.20–0.59)	<0.001	0.36 (0.20–0.65)	0.001
				0.79 (0.39–1.60)	0.519**

OR, odds ratio; CI, confidence interval; eGFR, estimated glomerular filtration rate (mL/min/1.73 m^2^); UACR, urinary albumin-to-creatinine ratio (mg/g)

*Age, sex, diabetes, hypertension, current smoking, atrial fibrillation, previous stroke, and alcohol history were adjusted in the multivariate analysis. In the multivariate model for the UACR (albuminuria), the eGFR was additionally adjusted. In the multivariate model for the eGFR, the UACR was additionally adjusted.

** National Institutes of Health Stroke Scale score was additionally adjusted

## Discussion

Renal dysfunction (decreased eGFR or albuminuria) is an independent risk for stroke and other cardiovascular events in the general population [8, 9]. In addition, some studies have demonstrated a significant association between renal dysfunction and adverse clinical outcomes, including mortality or stroke recurrence in patients who have suffered a stroke [2–7]. Of these, several studies included both of the two renal biomarkers in their analyses for mortality and functional outcome after stroke [3–7]. The results of those studies mostly indicated that albuminuria (or proteinuria), but not eGFR, is closely associated with the outcome after stroke [3–6].

In line with those previous studies, albuminuria was independently associated with mortality and major vascular events while eGFR was not in our study. In addition, albuminuria seemed more closely associated with good functional outcome at 6 months than eGFR though its association disappeared when NIHSS score was additionally adjusted. Thus, albuminuria seems a more useful clinical indicator than eGFR in predicting the risk of adverse outcome after stroke. In fact, the greater magnitude of association of vascular risk with albuminuria than with eGFR was recently demonstrated in a meta-analysis of 24 general population cohorts without a history of cardiovascular disease, which showed that eGFR and UACR improve the discrimination of cardiovascular outcomes beyond traditional risk factors, but the improvement was greater with UACR than with eGFR [8].

The mechanism by which albuminuria is related to post-stroke outcome is unclear, but there are several plausible explanations. First, albuminuria reflects systemic endothelial dysfunction with increased vascular permeability, which leads to an accumulation of atherogenic particles within arterial walls facilitating progressive atherosclerosis [7, 16, 17]. It may also increase the probability of symptomatic hemorrhagic transformation in an ischemic stroke lesion resulting in a worse outcome [17, 18]. Therefore, albuminuria may not only be associated with risk for ischemic stroke, but also risk for hemorrhagic stroke [9].

Moreover, albuminuria may be a marker for the acute phase reaction under stroke-related systemic inflammation and has been correlated with interleukin-6 level [19], which is associated with stroke lesion severity and worse outcomes [20]. Our results also showed, similar to findings of other studies [5, 6], that initial stroke severity (NIHSS score) was more increased in patients with albuminuria than in patients without albuminuria. Therefore, albuminuria may reflect an increased inflammatory reaction caused by severe stroke.

In addition, albuminuria is reportedly related to hemostatic abnormality, thereby facilitating thrombotic propensity in patients with stroke, followed by a worse outcome [21–23].

Our study had some limitations. First, it was based on a single center data with a small sample size;107 patients were excluded from the study due to the absence of urine albumin or 6-month outcome data. It thus can be limited by selection bias. In this regard, our results may not be generalizable to other stroke populations, and a large-scale prospective study is needed to verify our conclusions. Second, no information about premorbid renal status was available in our cohort, and urine albumin was only once measured after stroke. Accordingly, we could not determine if the detected urine albumin reflected premorbid impaired renal status or renal change reactive to systemic inflammation associated with an index stroke event. In addition, we excluded 12 patients who were undergoing dialysis but represented severely impaired renal dysfunction. However, our study objective was to compare the clinical significance of the two renal biomarkers, and the proportion of patients undergoing dialysis was so low (4.1%) as not to affect our analysis. Last, this was an observation study, so we could not control for all the differences between the groups though trying to adjust potential confounders (residual confounding).

The strength of our study lies in simultaneous measurement of eGFR and albuminuria and long-term monitoring of vascular events and functional outcomes, including mortality. In fact, to our best knowledge, this is the first study based on a stroke population to compare the two renal biomarkers through long-term monitoring of both vascular events and mortality.

Collectively, our results showed that albuminuria, but not eGFR, was significantly associated not only with mortality but also with further vascular events in patients with ischemic stroke. Therefore, albuminuria seems a more useful clinical indicator than eGFR in evaluating the risk of adverse outcome after stroke.
